# Motor competence and compliance with physical activity recommendations in Chilean schoolchildren

**DOI:** 10.3389/fpsyg.2024.1371766

**Published:** 2024-05-10

**Authors:** Nicolas Martinez-Lopez, Isaac Estevan, Paulina Candia-Cabrera, Nicolas Aguilar-Farias, Jaime Carcamo-Oyarzun

**Affiliations:** ^1^CIAM Physical Literacy Research Centre, Faculty of Education, Social Science, and Humanities, Universidad de La Frontera, Temuco, Chile; ^2^Programa de Doctorado en Didácticas Especificas, Didáctica de la Educación Física, University of Valencia, Valencia, Spain; ^3^Activitat Física i Promoció de la Salut (AFIPS) Research Group, Department of Teaching of Physical Education, Arts, and Music, University of Valencia, Valencia, Spain; ^4^Department of Physical Education, Universidad de La Frontera, Temuco, Chile; ^5^UFRO Activate Research Group, Universidad de La Frontera, Temuco, Chile

**Keywords:** motor development, motor skills, motor competence, physical activity, physical literacy

## Abstract

The development of motor competence is thought to be a crucial precursor to raising the trajectory of PA throughout a person’s life. The objectives of this study are to determine motor competence and the daily time of moderate and vigorous physical activity of students in 5th and 6th grade elementary in Chile, and to establish whether there are differences in motor competence according to sex and compliance with physical activity recommendations. 368 schoolchildren (*M* = 11.10 years; 54.3% girls) participated. To assess motor competence, the MOBAK 5–6 test was used. Physical activity was measured using ActiGraph wGT3X-BT® accelerometers. Boys (*M* = 3.65, SD = 2.14) showed better performance than girls (*M* = 2.39, SD = 1.80) in Object Control (*p* ≤ 0.001, PS = 0.67). For Self-Movement, the girls (*M* = 2.72, SD = 2.14) performed better than the boys (*M* = 2.40, SD = 1.86); however, there were no significant differences between the two sexes (*p* = 0.257). Boys (*M* = 48.4, SD = 22.8) presented more daily minutes of moderate and vigorous physical activity than girls (*M* = 35.9, SD = 16.9), with statistically significant differences (*p* ≤ 0.001, PS = 0.67). About MC according to compliance with the physical activity recommendations, only in Object Control there is a statistically significant difference (*p* ≤ 0.001; PS = 0.29) between the students who comply with the recommendations (*M* = 4.28, SD = 2.12) and those who do not achieve the recommended minutes (*M* = 2.67, SD = 1.29). By contrast, an analysis of Self-Movement found no significant difference (complies: *M* = 2.73, SD = 1.97; does not comply: *M* = 2.54, SD = 2.04; *p* = 0.408) between the two groups. It is necessary to generate instances that develop motor competence in all its dimensions to promote higher levels of moderate to vigorous physical activity.

## 1 Introduction

Physical activity (PA) provides multiple benefits to people’s health (Telama et al., 2005); however, the number of people who comply with PA recommendations is very low (Hallal et al., 2012), including a large proportion of the child population (Aubert et al., 2022). Current worldwide PA recommendations for children and adolescents indicate that they should engage in an average of at least 60 min of mainly aerobic physical activity of moderate to vigorous intensity daily (World Health Organization, 2020), however, several studies indicate that low percentages of children and adolescents comply with these recommendations (Aubert et al., 2022). In the particular case of Chile, this situation is worse, as it is one of the countries with the worst PA-related indicators (Aubert et al., 2022). Through the use of accelerometers, several studies have objectively identified that the percentage of children and adolescents who comply with these recommendations ranges between 3.7 and 14.6% (Toledo-Vargas et al., 2020; Ceppi-Larraín et al., 2021). These figures show that sedentary behaviors can become entrenched among a high proportion of the Chilean child population if measures are not taken to address this issue (Aguilar-Farías et al., 2017). To reverse this problem, factors that favor the practice of regular PA need to be developed (Robinson et al., 2015), especially moderate and vigorous physical activity (MVPA) (Silva-Santos et al., 2021). One of these is motor competence (MC), which is thought to be a crucial precursor to raising the trajectory of PA throughout a person’s life (Stodden et al., 2008; Barnett et al., 2016; Tyler et al., 2020).

MC is defined as a person’s ability to dominate a variety of locomotor and stability skills, and control objects needed to carry out daily tasks (Utesch and Bardid, 2019). Theoretical models have suggested that MC interacts positively with PA (Stodden et al., 2008; Robinson et al., 2015), identifying MC as essential to a physically active lifestyle. Several empirical studies have confirmed this interaction based on these theoretical models, identifying a positive association between MC and PA (Holfelder and Schott, 2014; Logan et al., 2015; Lopes et al., 2020). In fact, PA and MC presented a reciprocal association across childhood and early adolescence (Lima et al., 2017).

Although the importance of developing MC in the child population is recognized, performance levels are concerning. In Europe, various studies have demonstrated that students have low MC, with a high proportion of students who require motor reinforcement (Herrmann et al., 2016; Quitério et al., 2017; Scheuer et al., 2019; Duncan et al., 2020; Wälti et al., 2022). In Latin America, particularly in Chile, the panorama is no different, and studies show low levels of MC (Martinez-Lopez et al., 2021; Rodríguez-Briceño et al., 2021; Müller et al., 2022; Quintriqueo-Torres et al., 2022; Carcamo-Oyarzun et al., 2023). Previous studies have already identified that most schoolchildren have gross motor development levels categorized as poor and very poor (Luarte et al., 2014; Bermúdez-Ferrales et al., 2015), which is why this situation has not changed in recent years. One important aspect to consider in MC development is the role of sex. There is strong evidence that boys present significantly higher levels in object control tasks, both in international (Iivonen and Sääkslahti, 2014; Barnett et al., 2016) and Chilean studies (Carcamo-Oyarzun and Herrmann, 2020; Martinez-Lopez et al., 2021; Rodríguez-Briceño et al., 2021). However, in tasks related to self-movement, there is no certain consensus, as in some studies the results indicate that girls perform better than boys (Iivonen and Sääkslahti, 2014), while in others no differences have been found between both sexes (Barnett et al., 2016). In studies within the Chilean context, the trend indicates that in the performance of boys and girls in self-movement skills there are no significant differences (Rodríguez-Briceño et al., 2021; Quintriqueo-Torres et al., 2022; Gonzalez-Huenulef et al., 2023).

Although previous studies have demonstrated the link between MC and PA (Lopes et al., 2020), further study is still needed to better understand this interaction (Barnett et al., 2022). Few studies have addressed this topic from the point of view of compliance with child physical activity recommendations; hence, this study has two purposes: (a) to determine motor competence and the daily time of moderate and vigorous physical activity of students in 5th and 6th grade elementary in Chile, and (b) to establish whether there are differences in motor competence according to sex and compliance with physical activity recommendations.

## 2 Method

### 2.1 Participants

A non-probability sample by convenience comprised 368 elementary students from 5th and 6th grade (*M* = 11.10, DE = 0.91 years; 54.3% girls) from five schools in la región de la Araucanía, Chile. The inclusion criteria were regular participation in physical education classes and having no health problems that would prevent them from performing the tests. All the participants had signed permission from their fathers, mothers, or guardians. In addition, each participant indicated their intention to participate by signing a consent form. The Universidad de La Frontera Scientific Ethics Committee approved the study protocol, file 125_17.

### 2.2 Instruments

#### 2.2.1 Evaluation of motor competence

To assess MC, the MOBAK 5–6 test was used, which was created by Herrmann and Seelig (2017) and validated in Spanish by Carcamo-Oyarzun and Herrmann (2020). This test focuses on the resolution of 8 motor skills tasks consistent with the curricular objectives of the subject of physical education (Carcamo-Oyarzun et al., 2022). The structure of the test groups these tasks in 2 dimensions: (a) Object Control, corresponding to four tasks associated with motor manipulative skills (bouncing, dribbling, throwing, and catching), and (b) Self-Movement, comprised of four tasks linked to locomotor and stability skills (jumping, running, rolling, and balancing). Table 1 describes each of the tasks on the MOBAK 5–6.

**Table 1:** Descriptive summary of the tasks on the MOBAK 5–6 Test [adapted from Carcamo-Oyarzun and Herrmann (2020)].

Dimension	Task	Description
Object control	Throwing	Throw a ball to try to hit a circle marked on a wall from 3.5 m away.
	Catching	Throw a tennis ball against a wall and then catch it in the air.
	Bouncing	Bounce a size 6 basketball along a track (8.0 m x 1.1 m) with 4 obstacles.
	Dribbling	Dribble a size 4 indoor football along a track (8.0 m x 1.1 m) with 4 obstacles.
Self-movement	Balancing	Walk forwards and then back on a balanced upside-down bench with 2 obstacles 12 cm high.
	Rolling	Perform a forward somersault, starting off standing up and passing over a cardboard box.
	Jumping	Jump rope for 20 s., changing pace or form at 10 s.
	Running	Running forwards and diagonally in a square (4.0 m x 4.0 m), taking 3 steps over gymnastics hoops when running forwards.

There are 2 attempts for each motor task, except throwing and catching, where 6 attempts are included for each. The score is dichotomous (0 = unsuccessful, 1 = achieved). Therefore, a record of valid attempts must be kept considering that 0 times achieved is 0 points, 1 time achieved is 1 point, and 2 times achieved is 2 points. In the case of throwing and catching, considering there are 6 attempts, the score corresponds to 0 points when 0 to 2 times are achieved, 1 point for 3 to 4 times, and 2 points for 5 to 6 successful attempts. Then, the sum of the scores obtained in each task gives the score of the respective dimension, with a possible score of a minimum of 0 points and a maximum of 8 points. The application details and scoring of the MOBAK 5–6 test can be found at https://www.mobak.cl/mobak-5-6.

#### 2.2.2 Daily time of moderate and vigorous physical activity

To measure the amount of MVPA, the ActiGraph wGT3X-BT accelerometer was used, a device that records continuous and high-resolution data of physical activity for 24 h a day. The data were downloaded with a storage time of 60 s using the ActiLife 6.13.4 software. First, sleep was detected with the options Scoring Sleep and Detection Sleep to conduct an individual review to identify possible inconsistencies. Subsequently, the time of use was validated with the Choi algorithm, and it was stipulated that for the use of the accelerometer to be valid, it had to be used 4 days, where at least 1 must be a weekend and with a minimum of 10 h of correct use per day. Finally, the intensity of the physical activity was determined with the Evenson et al. (2008) algorithm. For a student to be included in the category of compliance with the physical activity recommendations, the physical activity recommendations proposed by the World Health Organization for children and adolescents between 5 and 17 years of age were taken as the baseline, consisting of 60 min of moderate to vigorous intensity physical activity per day (World Health Organization, 2020).

### 2.3 Procedure

The participants were asked to wear the accelerometer constantly for 7 days straight (Tudor-Locke et al., *2015*). The accelerometers were programmed with a storage time of 60 s (epochs), 4 days of minimum use with at least 1 day on a weekend, considering 10 h of use adequate for a day to be valid, stipulating its use on the right side of the waist. Then, an accelerometer was delivered to each student at school with its respective code. The correct way to use it was explained, indicating that they must keep the device on for 7 days, including sleeping, and they can only take it off for showering, swimming, or any activity that would get the strap wet. Once they had their accelerometer, the students moved to the gymnasium to take the MOBAK 5–6 test, where trained evaluators had prepared each of the 8 motor tasks. Each evaluator was in charge of 3 to 5 students depending on the number of students in each course. Each motor task was first explained and then demonstrated. An approximate time between 45 and 60 min was considered to finish evaluating the entire group.

### 2.4 Statistical analysis

The data analysis was performed using SPSS Statistics 25.0. Descriptive analyses of central tendency were done. To determine the normality of the sample, the Kolmogorov–Smirnov test was used. After identifying that the sample presents a non-normal distribution, the Mann–Whitney U test was used to analyze the differences in motor competence based on sex and compliance with the physical activity recommendations. *p* < 0.05 was considered the level of significance. The probability of superiority (PS) was calculated to determine the effect size, an analysis recommended when using non-parametric tests that seek comparisons of two groups (Li, 2016). The interpretation corresponded to PS ≥ 0.56 as a small effect size, PS ≥ 0.64 as medium, and PS ≥ 0.71 as large (Grissom, 1994). To analyze whether there are sex differences between students who comply with the recommendations and those who do not, a nonparametric Kruskal-Wallis test was used.

## 3 Results

Table 2 provides the descriptive analyses of the motor tasks of the MOBAK 5–6 test and minutes of MVPA per day according to the student’s sex. In terms of MC, for Object Control, the boys (*M* = 3.65, SD = 2.14) performed better than the girls (*M* = 2.39, SD = 1.80), being statistically different (*p* ≤ 0.001, PS = 0.67). For Self-Movement, girls (*M* = 2.72, SD = 2.14) and boys (*M* = 2.40, SD = 1.86) exhibited similar values (*p* = 0.257). In terms of daily time of MVPA, the boys (*M* = 48.4, SD = 22.8) recorded more minutes than the girls (*M* = 35.9, SD = 16.9), with statistically significant differences (p ≤ 0.001, PS = 0.67). Both groups’ mean MVPA daily times are below the recommended 60 min daily that the World Health Organization suggests.

**Table 2:** Descriptive statistics of motor tasks on the MOBAK 5–6 test and minutes of MVPA per day, based on sex.

	Girls (*n* = 200)			Boys (*n* = 168)			*U*	*p*	PS
	Mean (SD)	95% CI	Median	Mean (SD)	95% CI	Median			
**Motor competence**									
*Throwing^i^*	0.42 (0.59)	[0.34–0.50]	0.0	0.61 (0.68)	[0.50–0.72]	0.5	19267.0	0.006*	0.57
*Catching^i^*	0.34 (0.65)	[0.25–0.43]	0.0	0.77 (0.83)	[0.64–0.89]	1.0	21586.5	0.000*	0.64
*Bouncing^i^*	1.12 (0.84)	[1.00–1.24]	1.0	1.36 (0.78)	[1.24–1.48]	2.0	19426.0	0.005*	0.58
*Dribbling^i^*	0.51 (0.74)	[0.40–0.61]	0.0	0.90 (0.84)	[0.78–1.03]	1.0	21198.0	0.000*	0.63
Total object control^ii^	2.39 (1.80)	[2.13–2.64]	2.0	3.65 (2.14)	[3.32–3.97]	4.0	22500.5	0.000*	0.67
*Balancing^i^*	0.81 (0.81)	[0.69–0.92]	1.0	0.71 (0.81)	[0.58–0.83]	0.0	15660.0	0.225	-
*Rolling^i^*	0.58 (0.83)	[0.46–0.69]	0.0	0.58 (0.85)	[0.45–0.71]	0.0	16780.0	0.981	-
*Jumping^i^*	0.52 (0.77)	[0.41–0.62]	0.0	0.22 (0.52)	[0.14–0.30]	0.0	27883.0	0.000*	0.83
*Running^i^*	0.83 (0.84)	[0.71–0.94]	1.0	0.89 (0.84)	[0.76–1.02]	1.0	17479.0	0.474	-
Total self-movement^ii^	2.72 (2.14)	[2.42–3.02]	2.0	2.40 (1.86)	[2.11–2.68]	2.0	15662.6	0.257	-
**Moderate to vigorous physical activity per day**									
MVPA^iii^	35.9 (16.9)	[33.56–38.27]	32.5	48.4 (22.8)	[44.93–51.90]	46.0	22489.0	0.000*	0.67

^i^Range of 0 to 2 pts. ^ii^Range of 0 to 8 pts. ^iii^Minutes daily.

**Figure 1:**
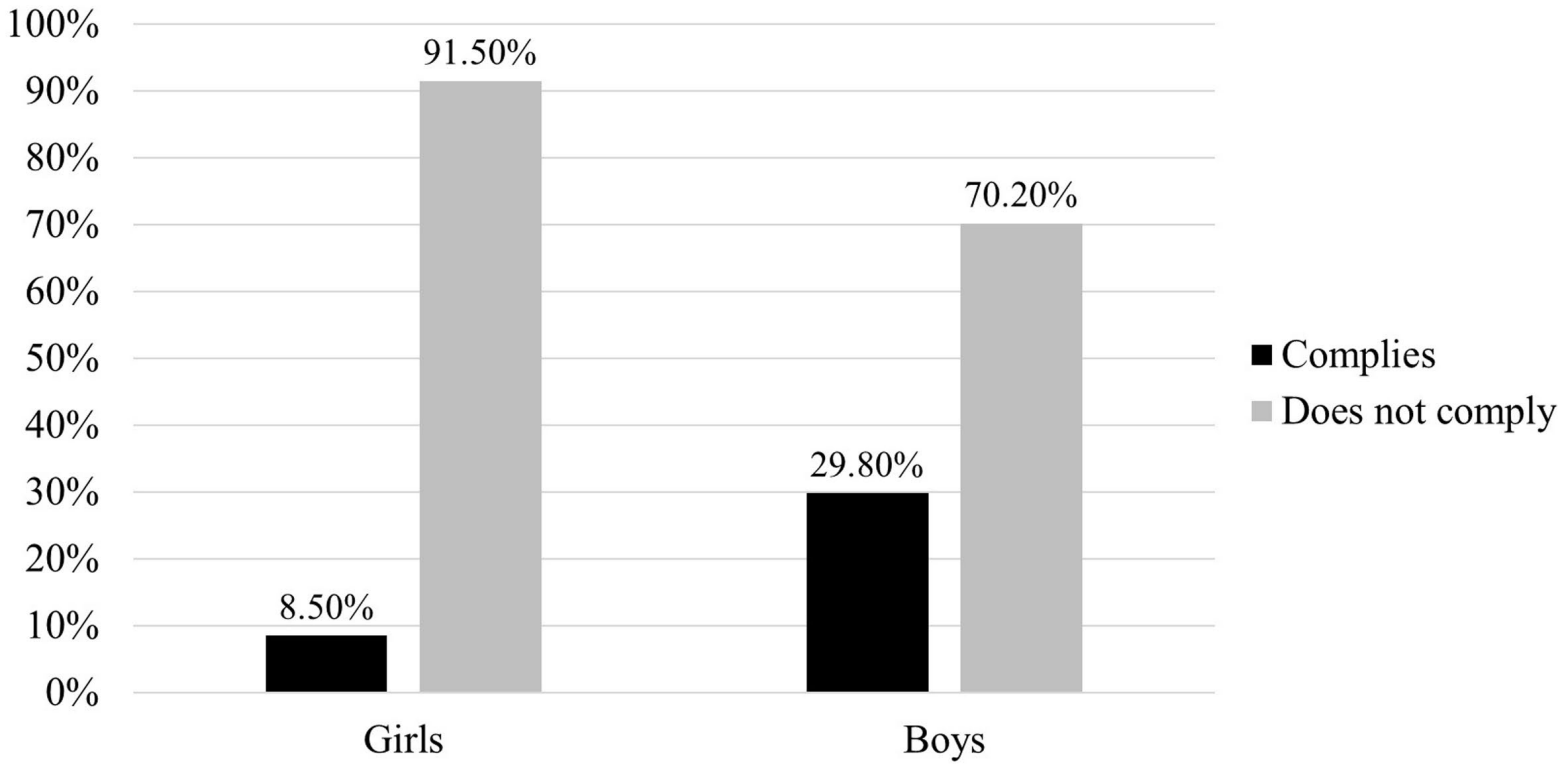
Compliance with the physical activity recommendations according to sex.

Figure 1 illustrates the percentages of compliance with the WHO’s physical activity guidelines. 29.8% of the boys complied with the 60 min of daily MVPA, a much higher percentage than the girls, as in this group, only 8.5% managed to achieve the recommended PA levels.

Table 3 describes the results in MC according to compliance with PA recommendations. In Object Control, there is a significant difference (*p* ≤ 0.001; PS = 0.29) between the students who comply with the recommendations (*M* = 4.28, SD = 2.12) and those who do not achieve the recommended minutes (*M* = 2.67, SD = 1.29). By contrast, an analysis of Self-Movement found no significant difference (complies: *M* = 2.73, SD = 1.97; does not comply: *M* = 2.54, SD = 2.04; *p* = 0.408) between the two groups.

**Table 3:** Differences in motor tasks on the MOBAK 5–6 test based on compliance with physical activity recommendations.

	Complies (*n* = 67)			Does not comply (*n* = 301)			*U*	*p*	PS
	Mean (SD)	95% CI	Median	Mean (SD)	95% CI	Median			
*Throwing^i^*	0.73 (0.73)	[0.55–0.91]	1.0	0.46 (0.61)	[0.39–0.53]	0.0	8066.5	0.004*	0.40
*Catching^i^*	1.03 (0.89)	[0.81–1.25]	1.0	0.43 (0.69)	[0.35–0.50]	0.0	6356.5	0.000*	0.32
*Bouncing^i^*	1.48 (0.75)	[1.30–1.66]	2.0	1.18 (0.83)	[1.08–1.27]	1.0	8074.0	0.006*	0.40
*Dribbling^i^*	1.04 (0.86)	[0.83–1.25]	1.0	0.61 (0.78)	[0.52–0.70]	0.0	7286.5	0.000*	0.36
Total object control^ii^	4.28 (2.12)	[3.77–4.80]	5.0	2.67 (1.92)	[2.45–2.89]	2.0	5799.0	0.000*	0.29
*Balancing^i^*	0.72 (0.77)	[0.53–0.91]	1.0	0.77 (0.82)	[0.68–0.86]	1.0	10367.5	0.696	-
*Rolling^i^*	0.79 (0.90)	[0.57–1.01]	0.0	0.53 (0.82)	[0.44–0.62]	0.0	8506.5	0.018*	0.42
*Jumping^i^*	0.30 (0.60)	[0.15–0.45]	0.0	0.40 (0.70)	[0.32–0.48]	0.0	10676.5	0.331	-
*Running^i^*	0.93 (0.84)	[0.72–1.13]	1.0	0.84 (0.84)	[0.74–0.93]	1.0	9494.0	0.423	-
Total self-movement^ii^	2.73 (1.97)	[2.25–3.21]	2.0	2.54 (2.04)	[2.31–2.77]	2.0	9440.0	0.408	-

^i^Range of 0 to 2 pts. ^ii^Range of 0 to 8 pts.

**Figure 2:**
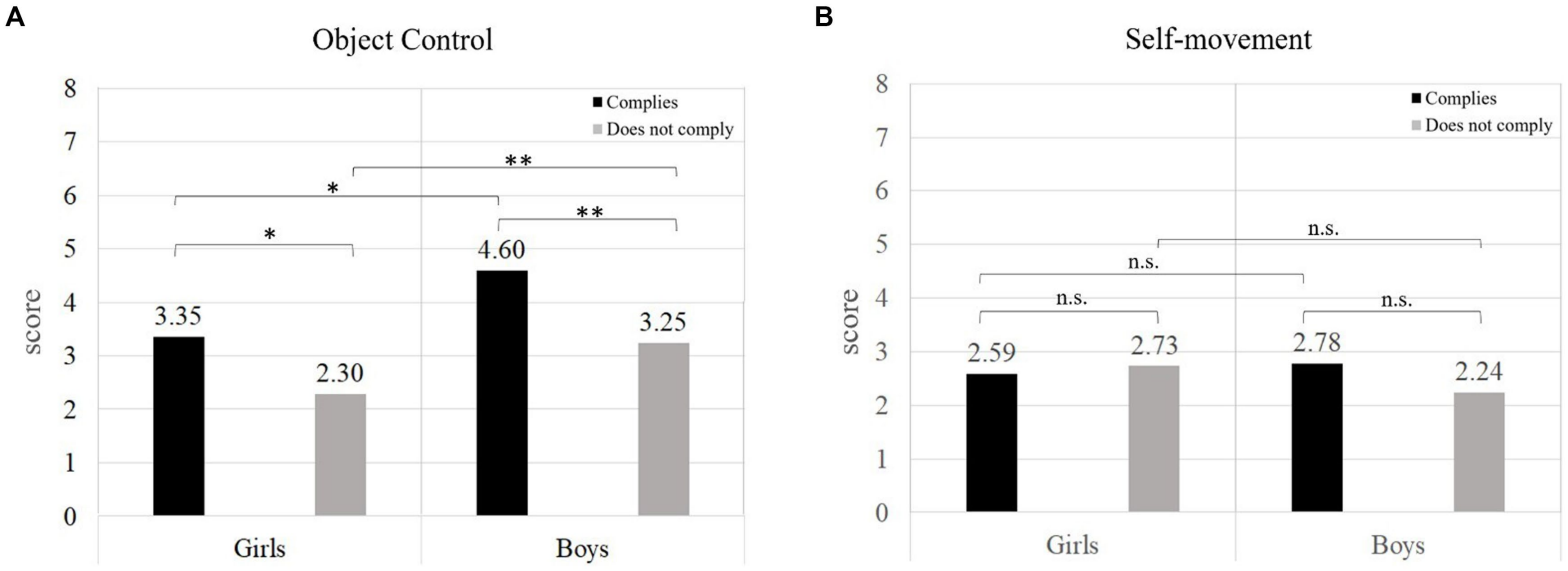
Comparison of motor competence according to sex between students who comply with the recommendations and those who do not. **(A)** Object control; **(B)** Self-movement. *significant differences at the level of *p* ≤ 0.05; **significant differences at the level of *p* ≤ 0.001; n.s., means no significant differences.

When analyzing whether there are differences according to sex between students who comply with the recommendations and those who do not, the results indicate that in Object Control there are significant differences (*H* = 48.925, df = 3, *p* < 0.001), specifically between boys and girls who comply (*p* = 0.036, PS = 0.67), boys and girls who do not comply (*p* < 0.001, PS = 0.64), girls who comply and girls who do not comply (*p* = 0.024, PS = 0.34), and boys who comply and boys who do not comply (*p* < 0.001, PS = 0.32) (Figure 2). Regarding the results of Self-Movement, no significant differences were found between the groups (*H* = 3.712, df = 3, *p* = 0.294).

## 4 Discussion

This study sought to analyze the MC and daily time of MVPA of 5th and 6th-grade elementary students in Chile and to establish if there are differences in MC based on sex and compliance with PA recommendations. The results referring to MC according to sex indicate that, in Object Control, there is a significant difference between the two groups, where the boys perform better than the girls, confirming this trend posited in international studies (Iivonen and Sääkslahti, 2014; Barnett et al., 2016; Brian et al., 2019) within the Chilean context (Carcamo-Oyarzun and Herrmann, 2020; Martinez-Lopez et al., 2021; Rodríguez-Briceño et al., 2021). In reference to the tasks associated with Self-Movement, girls obtained similar values than boys which is consistent with the findings of the systematic review by Barnett et al. (2016), where no differences or associations were determined according to sex in locomotor or stability tasks. Studies with a Chilean school population also agree with this tendency (Rodríguez-Briceño et al., 2021; Quintriqueo-Torres et al., 2022; Gonzalez-Huenulef et al., 2023). These differences could be explained by the existing stereotyping of physical activities, where the boys tend to participate in ball sports, whereas the girls participate in activities associated with body control (Crane et al., 2015; Temple et al., 2016).

With respect to the amount of MVPA that they do on average per day, the students analyzed reached a mean of 41.6 min of MVPA, below the times found in studies with Spanish students, who reached means between 53 and 56 min per day (Martínez Martínez et al., 2012; Calahorro-Cañada et al., 2015). In addition, the values of the Chilean students are below students in Australia (65 min per day) or Canada (58 min per day), although approaching students in India (48 min per day) and Brazil (44 min per day) (Thivel et al., 2019). When analyzing possible differences concerning sex, it is observed that boys perform more MVPA than girls, which coincides with other studies where boys are also found to be more active than girls (Glinkowska and Glinkowski, 2018; Gutierrez-Hervas et al., 2020; Ceppi-Larraín et al., 2021). This situation could be due to multiple factors, both biological, such as the structure of the muscle cells induced by sex hormones (Wang et al., 2019), and cultural, where boys receive more support and opportunities than girls for participating in certain physical activities (Temple et al., 2016).

Regarding the proportion of schoolchildren who meet the PA recommendations proposed by the WHO (60 min daily with moderate to vigorous intensity), in the sample studied, 18.2% reach this figure, which is higher than previous studies on the Chilean population, where it was identified that only 10.5% comply with this recommendation (Aguilar-Farias et al., 2021). Even so, these proportions show that most children in Chile do not comply with the current directives on PA, making it necessary to produce strategies that promote the factors associated with regular participation in physical activities (Guthold et al., 2020). Considering that MC is recognized as a factor that interacts bidirectionally with participation in PA (Barnett et al., 2022) when analyzing the MC results in terms of compliance with PA recommendations, it is noted that in Object Control, the children who comply with the PA recommendations show better results than the rest, with a significant difference. This situation is confirmed in comparisons by sex, where girls and boys who comply with PA recommendations differ significantly from their respective peers who do not comply. The results in Object Control agree with this MC-PA bidirectionality (Barnett et al., 2022); however, with respect to Self-Movement, although the students who comply with the PA recommendations have slightly higher values than those who do not, these differences are not statistically significant.

When the interaction between sex and PA recommendations was analyzed, interesting findings were obtained in object control skills. Boys and girls who comply with PA recommendations outperformed their peers who do not comply. These results underscore the need to offer programs to increase regular participation in PA, which includes activities that enable the development of the two dimensions of MC. Several studies have identified that the students who participate in physical-sport workshops outside school do more PA and present better indicators in their MC (Strotmeyer et al., 2020; Müller et al., 2022; Wälti et al., 2022; Carcamo-Oyarzun et al., 2023). Thus, the results of this study serve as input for teachers on which to base their methodological strategies oriented to the development of MC. At the same time, schools can promote activities that offer opportunities for their students to have various options for PA, like extracurricular workshops focusing on developing MC. Moreover, we cannot leave out the promotion of public policies where the aforementioned measures are reinforced to strengthen the development of schoolchildren and generate active and healthy lifestyles over time. These results are important not only because they add to the limited evidence that exists in Latin America on this issue, but also because they highlight the need to take measures to promote physical activity and the development of physical literacy in this region (Ortega-Benavent et al., 2024).

Despite the relevant findings presented, this study is not without limitations. It is possible to mention that the study design, being cross-sectional, does not determine causal links between MC and PA. Another limitation is that it must be borne in mind that the number of schoolchildren who comply with PA recommendations is very low, which is why the differences encountered must be considered with caution. Furthermore, this study did not consider the type of PA that students develop or its context, which could have given more robust information concerning the results of MC based on compliance with PA recommendations. However, it is also important to highlight the strengths of the study, which lie in the instruments used for data collection. The measurement of MVPA time using accelerometers stands out, providing much more objective data than other assessment tools, such as self-reported questionnaires. On the other hand, it is also important to point out that the evaluation of MC has been performed previously with a test validated for the school population participating in this study. For future studies, we recommend considering other intensities in addition to MVPA, and even considering compositional and isotemporal analyses, to see if increasing a certain number of minutes of MVPA at the expense of sedentary or light PA can help to achieve the minimum recommendations. In addition, we recommend to use longitudinal designs to understand the bidirectionality in the interaction between MC and PA, as well as the inclusion of underlying variables that act as mediators in this association, such as perceived MC and health-related physical conditioning.

In conclusion, in this study, differences were found in MC according to the sex of the students, specifically in the dimension Object Control, where the boys present a better motor performance in these tasks than the girls. By contrast, no significant differences were noted between the two groups in the dimension Self-Movement. Regarding the time of MVPA, the analyses revealed differences between boys and girls, with the boys presenting more minutes of daily activity in this range of intensity. In addition, most children do not manage to reach to complete the recommendations of 60 min daily of MVPA. In that sense, the children who comply with the recommendations of 60 min of MVPA daily present higher MC values in the dimension Object Control than those who do not, whereas in the dimension Self-Movement, the two groups do not differ. This highlights the need to implement strategies that develop MC and promote PA programs both at school and beyond, offering more alternatives for participation that can stimulate adherence to the regular practice of PA.

## Data availability statement

The raw data supporting the conclusions of this article will be made available by the authors, without undue reservation.

## Ethics statement

The studies involving humans were approved by the Universidad de La Frontera Scientific Ethics Committee. The studies were conducted in accordance with the local legislation and institutional requirements. Written informed consent for participation in this study was provided by the participants’ legal guardians/next of kin.

## Author contributions

NM-L: Conceptualization, Formal analysis, Methodology, Writing – original draft, Writing – review & editing. IE: Conceptualization, Methodology, Supervision, Writing – original draft, Writing – review & editing. PC-C: Writing – original draft, Writing – review & editing. NA-F: Methodology, Writing – original draft, Writing – review & editing. JC-O: Conceptualization, Formal analysis, Funding acquisition, Methodology, Supervision, Writing – original draft, Writing – review & editing.
